# Prevalence of Monogenic Bone Disorders in a Dutch Cohort of Atypical Femur Fracture Patients

**DOI:** 10.1002/jbmr.4801

**Published:** 2023-04-19

**Authors:** Wei Zhou, Jeroen GJ van Rooij, Denise M van de Laarschot, Zografia Zervou, Hennie Bruggenwirth, Natasha M Appelman‐Dijkstra, Peter R Ebeling, Serwet Demirdas, Annemieke JMH Verkerk, M Carola Zillikens

**Affiliations:** ^1^ Department of Internal Medicine Erasmus MC Rotterdam The Netherlands; ^2^ Department of Clinical Genetics Erasmus MC Rotterdam The Netherlands; ^3^ Department of Internal Medicine, Division of Endocrinology Leiden University Medical Center Leiden The Netherlands; ^4^ Department of Medicine School of Clinical Sciences, Monash University Clayton Australia

**Keywords:** GENETICS, MONOGENIC BONE DISORDER, ATYPICAL FEMUR FRACTURES, OSTEOPOROSIS, WHOLE‐EXOME SEQUENCING, COPY NUMBER VARIATIONS, BISPHOSPHONATES

## Abstract

Atypical femur fractures (AFFs), considered rare associations of bisphosphonates, have also been reported in patients with monogenic bone disorders without bisphosphonate use. The exact association between AFFs and monogenic bone disorders remains unknown. Our aim was to determine the prevalence of monogenic bone disorders in a Dutch AFF cohort. AFF patients were recruited from two specialist bone centers in the Netherlands. Medical records of the AFF patients were reviewed for clinical features of monogenic bone disorders. Genetic variants identified by whole‐exome sequencing in 37 candidate genes involved in monogenic bone disorders were classified based on the American College of Medical Genetics and Genomics (ACMG) classification guidelines. Copy number variations overlapping the candidate genes were also evaluated using DNA array genotyping data. The cohort comprises 60 AFF patients (including a pair of siblings), with 95% having received bisphosphonates. Fifteen AFF patients (25%) had clinical features of monogenic bone disorders. Eight of them (54%), including the pair of siblings, had a (likely) pathogenic variant in either *PLS3*, *COL1A2*, *LRP5*, or *ALPL*. One patient carried a likely pathogenic variant in *TCIRG1* among patients not suspected of monogenic bone disorders (2%). In total, nine patients in this AFF cohort (15%) had a (likely) pathogenic variant. In one patient, we identified a 12.7 Mb deletion in chromosome 6, encompassing *TENT5A*. The findings indicate a strong relationship between AFFs and monogenic bone disorders, particularly osteogenesis imperfecta and hypophosphatasia, but mainly in individuals with symptoms of these disorders. The high yield of (likely) pathogenic variants in AFF patients with a clinical suspicion of these disorders stresses the importance of careful clinical evaluation of AFF patients. Although the relevance of bisphosphonate use in this relationship is currently unclear, clinicians should consider these findings in medical management of these patients. © 2023 The Authors. *Journal of Bone and Mineral Research* published by Wiley Periodicals LLC on behalf of American Society for Bone and Mineral Research (ASBMR).

## Introduction

Bisphosphonates are widely prescribed antiresorptive drugs, proven to be highly effective in preventing fragility fractures in older individuals.^(^ ^1^ ^)^ Atypical femur fractures (AFFs) are a rare type of fractures associated with bisphosphonate use, occurring with no or minimal trauma at the femoral shaft from the subtrochanteric region to just above the supracondylar flare.^(^ ^2^ ^)^ They can be distinguished from typical osteoporotic fractures by specific radiological features, such as a horizontal fracture line originating at the lateral side, no or minimal comminution, and localized cortical thickening.^(^ ^3^ ^)^

The incidence of AFFs is estimated to vary between 3 and 17 per 100,000 person‐years in the overall population and increases to 55 per 100,000 person‐years among patients with more than 3 years of bisphosphonate use.^(^ ^3^, ^4^, ^5^ ^)^ Although rare, these debilitating fractures have been of great concern for both patients and physicians and have contributed to a 50% decline in the use of these drugs in the US between 2008 and 2012.^(^ ^6^, ^7^ ^)^ This has led to various studies aiming to unravel the pathogenesis of AFFs, which is likely to be different from that of typical osteoporotic fractures.^(^ ^3^ ^)^ It has been suggested that AFFs are stress or insufficiency fractures,^(^ ^3^ ^)^ possibly resulting from accumulation of microdamage due to suppressed bone remodeling and/or increased homogeneity of bone mineralization caused by long‐term bisphosphonate use.^(^ ^8^ ^)^ Nevertheless, it remains unexplained why AFFs occur in only a minority of patients treated with bisphosphonates. Neither has it been explained why these fractures also occur in patients who never used bisphosphonates.^(^ ^4^, ^9^, ^10^ ^)^

Genetic components may contribute to the AFF pathogenesis, which is supported by several arguments, including greater risk for AFFs in Asians than Europeans^(^ ^11^ ^)^ and the occurrence of AFF in families^(^ ^12^ ^)^ and in monogenic bone disorders.^(^ ^13^ ^)^ Monogenic bone disorders are rare and caused by genetic variants in a single gene, typically inherited in a Mendelian pattern but also occurring de novo.^(^ ^14^ ^)^ In a systematic literature search, we have previously retrieved 57 published AFF cases in seven different monogenic bone disorders, ie, hypophosphatasia (HPP), X‐linked hypophosphatemia, pycnodysostosis, osteopetrosis, osteoporosis pseudoglioma syndrome (OPPG), osteogenesis imperfecta (OI), and X‐linked osteoporosis.^(^ ^13^, ^15^ ^)^ Remarkably, 56% of these patients had never used bisphosphonates and 17% were diagnosed with the monogenic bone disorder only after the occurrence of an AFF.^(^ ^15^ ^)^ These findings suggest that AFFs, in part, might be a consequence of an underlying monogenic bone disorder that remains undetected in some cases.

Some candidate gene studies exploring genetic causes of AFF have investigated, eg, genetic variants in one or more candidate genes such as *ALPL*, *COL1A1*, *COL1A2*, and *SOX9* in a small number of patients (<20), identifying only two likely pathogenic variants in *COL1A2* and *ALPL*, respectively.^(^ ^16^, ^17^, ^18^ ^)^ Many other genes associated with monogenic bone disorders have not been investigated for the presence of variants in AFF cohorts. Moreover, it has not yet been fully explored whether and how often the occurrence of AFFs is related to monogenic bone disorders. We hypothesize that a significant fraction of AFF patients have underlying monogenic bone disorders. To test this, we investigated the prevalence of monogenic bone disorders in a Dutch AFF cohort of 60 patients by assessing clinical and genetic data. We performed hospital medical chart review of clinical information and whole‐exome sequencing (WES) on all 60 AFF patients to assess the presence and pathogenicity of rare variants in a reviewed list of candidate genes associated with monogenic bone disorders. Additionally, we studied the presence of copy number variations by DNA by investigating single‐nucleotide polymorphism (SNP) array genotyping data.

## Materials and Methods

### Patient population

The patients included in this study were patients who experienced an AFF and were recruited at two specialist bone centers in the Netherlands, namely the Bone Center of Erasmus Medical Center (Erasmus MC, *n* = 53), Rotterdam, and the Center for Bone Quality from the Leiden University Medical Center (LUMC, *n* = 7), Leiden, including referrals from other hospitals in the Netherlands to these centers. All patients signed written informed consent to provide blood samples for genetic research. The study was approved by the Medical Ethical Committee of Erasmus MC under number MEC‐2013‐264.

The radiological features of all of the included AFF patients fulfilled the revised case definition for AFF published in the second American Society for Bone and Mineral Research (ASBMR) Task Force Report in 2014.^(^ ^3^ ^)^

For this study, the patients were examined by the treating physician MCZ and NMAD themselves or under their supervision, and medical files were reviewed to identify clinical suspicion of monogenic bone disorders by using known clinical features suggestive of these disorders as listed in Table 1.^(^ ^19^, ^20^, ^21^, ^22^, ^23^, ^24^ ^)^ Patients with clinical suspicion of a monogenic bone disorder were indexed with numbers starting with an “S” and those without were indexed with numbers starting with “NS.”

**Table 1:** Clinical Features of Monogenic Bone Disorders With Increased Fracture Risk

General features for monogenic bone disorders	Early‐age (<18 years) low‐trauma fractures
	A history of multiple low‐trauma fractures
	Severe osteoporosis not explained by secondary factors
	Family history of a monogenic bone disorder/osteoporosis
Signs of osteogenesis imperfecta	Blue or gray sclera
	Hyperlaxity of skin or joints
	Muscle weakness
	Hearing loss
	Discolored/brittle teeth
	Skeletal deformity
	Scoliosis
	Joint pain
	Short stature
Hypophosphatemia	Serum phosphate level decreased below the lower level of reference levels (for age) in combination with a decreased tubular reabsorption of phosphate
	Excluding nongenetic cause for hypophosphatemia
	With or without a positive family history
Hypophosphatasia	Serum level of alkaline phosphatase (ALP) repeatedly below 40 U/L with increased serum levels of pyridoxal 5′‐phosphate (PLP)
	With or without increased excretion of phosphoethanolamine (PEA) in urine
	Excluding other causes for low ALP

In clinical practice, genetic testing for a monogenic bone disorder was performed based on the evaluation by the treating physician and multidisciplinary team and only if the patient consented. The testing included massive parallel sequencing gene panels or Sanger sequencing as indicated in Supplemental Table S1.

### Candidate gene selection

We assessed the presence and the pathogenicity of variants detected by WES in a reviewed list of 37 candidate genes known to be involved in monogenic bone disorders. In patients who were already genetically tested with a gene panel or Sanger Sequencing, WES was performed to confirm the presence of variants or identify variants that might have been missed by tested gene panels. The 37 candidate genes were selected from the literature and OMIM, and presented in Supplemental Table S2. This list includes all genes as presented in a paper on Mendelian bone fragility disorder from Robertson and Rauch,^(^ ^25^ ^)^ as well as previously reported genes with a pathogenic variant in AFF patients,^(^ ^15^ ^)^ or tested for OI and related diseases (v2) by the VUmc (Amsterdam UMC, the Netherlands) and supplemented with genes involved in juvenile osteoporosis.

### Whole‐exome sequencing analysis

WES was performed on DNA of all 60 AFF patients regardless of prior diagnostic genetic testing in clinical practice. DNA was isolated from peripheral blood by the Human Genomics Facility (HuGeF), Department of Internal Medicine, Erasmus MC, using a standard protocol as described before.^(^ ^26^ ^)^ The DNA library was constructed using the KAPA library preparation kit (Roche Diagnostics, Inc, Pleasanton, CA, USA). Exome capture was performed using the Nimblegen SeqCap EZ MedExome Capture Kit (Roche Nimblegen, Inc, Madison, WI, USA). Paired‐end reads (2 × 150 bp) sequenced with the Illumina (San Diego, CA, USA) NovaSeq 6000 platform were demultiplexed and aligned to the human reference genome UCSC build hg19 using the Burrows‐Wheeler alignment tool (BWA version 0.7.3a). The Genome Analysis ToolKit (GATK version 3.8) was used for indel realignment and base quality score recalibration. Duplicates were marked with Picard Tools (version 2.18.4). HaplotypeCaller and GenotypeGVCFs (GATK 3.8) were used to generate per‐sample gVCF files and combined VCF file. The average depth of coverage per sample ranged between 57.7 and 141.9. For each of the 37 candidate genes, the percentage of bases in the exons covered at 20× and 30× is shown in Supplemental Table S2. Variants with QD score (QUAL score normalized by allele depth) ≤5 were filtered out.

All detected single nucleotide variants (SNVs) and indels were annotated using ANNOVAR (version 2019‐10‐24). Gene definition was based on RefSeq (NCBI Reference Sequence Database). We used allele frequencies from the 1000 Genome Project (version p3v5)^(^ ^27^ ^)^ and the Genome Aggregation Database (GnomAD) Exome and Genome data set (version 2.1.1).^(^ ^28^ ^)^ Predicted pathogenicity and conservation scores were obtained from SIFT,^(^ ^29^ ^)^ PolyPhen2,^(^ ^30^ ^)^ LRT, MutationTaster,^(^ ^31^ ^)^ FatHMM,^(^ ^32^ ^)^ RadialSVM,^(^ ^33^ ^)^ GREP++,^(^ ^34^ ^)^ and Combined Annotation Dependent Deletion (CADD), which integrates the other programs to generate an overall deleteriousness score.^(^ ^35^ ^)^ A CADD score ≥20 represents a prediction of the variant being among the 1% most deleterious in the genome. Additionally, the ClinVar database^(^ ^36^ ^)^ and the Leiden Open‐source Variation Database (LOVD)^(^ ^37^ ^)^ were referenced to aid in classification of variant pathogenicity.

### Filtering and classification of variants in WES data

We restricted the analysis to nonsynonymous, stopgain, stoploss, and splicing variants located in the exonic regions and exon flanking intronic regions, UTRs, and exonic indels in the 37 candidate genes (as listed in Supplemental Table S2). Variants were filtered based on the allele frequency in the overall population of the reference databases (1000 Genomes Project and GnomAD). Variants were filtered at an allele frequency cut‐off of 0.001 for the 15 genes associated with dominantly inherited bone disorders, ie, *COL1A1*, *COL1A2*, *IFITM5*, *SGMS2*, *P4HB*, *LRP5*, *WNT1*, *PLS3*, *CLCN7*, *PLEKHM1*, *TNFRSF11A*, *ALPL*, *PHEX*, *DKK1*, and *WNT3A*. Because the population frequency of one disease‐causing allele is not necessarily very low for a recessive disorder,^(^ ^38^ ^)^ variants were filtered at a less stringent allele frequency cut‐off of 0.01 for the remaining 22 genes associated with recessively inherited bone disorders. In the filtered list, we checked for the presence of heterozygous variants in dominant disease genes and homozygous variants or compound heterozygous variants in recessive disease genes. Variants were classified as pathogenic, likely pathogenic, variants of uncertain significance (VUS), likely benign, or benign using the American College of Medical Genetics and Genomics (ACMG) guideline for sequence variants interpretation.^(^ ^39^ ^)^

### Comparison with population controls and statistical analysis

WES data of controls were downloaded from gnomAD (v2; https://gnomad.broadinstitute.org/downloads/). We included variants in the 37 candidate genes that passed quality control (https://gnomad.broadinstitute.org/help/variant-qc). After applying the same variant filtering criteria as described above, 12,598 variants were left. As an alternative to manually classifying a large number of variants using the ACMG guideline, variants were classified by filtering. Variants were classified as (likely) pathogenic variants when they fulfilled the following criteria: (i) extremely rare (frequency <0.0001) in population database (ACMG criterion PM2); (ii) deleterious effect supported by computational evidence (CADD >15) (ACMG criterion PP3); and (iii) LOF variants (ACMG criterion PVS1) or previously established as a pathogenic variant (ACMG criterion PS1) or reported as likely pathogenic (ACMG criterion PP5) in Clinvar. (Likely) benign variants were identified by filtering for variants classified as (likely) benign in Clinvar. Other variants were regarded as VUS. All filtering steps are shown in Supplemental Table S5. Because the individual phenotype and genotype data of the controls were not available, the percentages of individuals with (likely) pathogenic variants and VUS were estimated by assuming that every carrier only had one of these variants. These percentages in controls were compared with the respective percentages in cases estimated using the same filtering criteria and assumptions with a binomial test.

### Genotyping and copy number variation (CNV) analysis

Genotyping was performed using the Infinium (Illumina) Global Screening Array (GSA) GSAMD‐v3. Signal intensity ratios (log R ratio [LRR]) and allelic frequencies (B allele frequency [BAF]) for each SNP were obtained with GenomeStudio 2.0 software (Illumina), without using a preexisting cluster file. Variants with a cluster separation score below 0.27 were filtered out. All samples had a call rate above 97.5%. Copy number deletions and duplications were investigated using both PENNCNV (version 2013‐02‐08)^(^ ^40^ ^)^ and Nexus Copy Number (version 10, BioDiscovery Inc, El Segundo, CA, USA) software. Copy number variations (CNVs) were filtered based on the presence of at least 10 consecutive SNPs per CNV and without a minimal size restriction. Only loci overlapping the candidate genes from Supplemental Table S2 were investigated for a possible CNV. Variants are described with genome positions in Genome Reference Consortium Human Build 37 (hg19). CNVs were classified according to the standards made jointly by the ACMG and the Clinical Genome Resource (ClinGen) and using the web application (https://cnvcalc.clinicalgenome.org/redmine/projects/cnvcalc/cnv_calculator/cnv-loss).^(^ ^41^ ^)^

## Results

In total, we analyzed WES data from 60 AFF patients recruited between September 2013 and April 2019, including 53 AFF patients from the Erasmus MC and 7 patients from the LUMC. Except for two siblings, all patients were unrelated.

### Patient characteristics

**Fig. 1:**
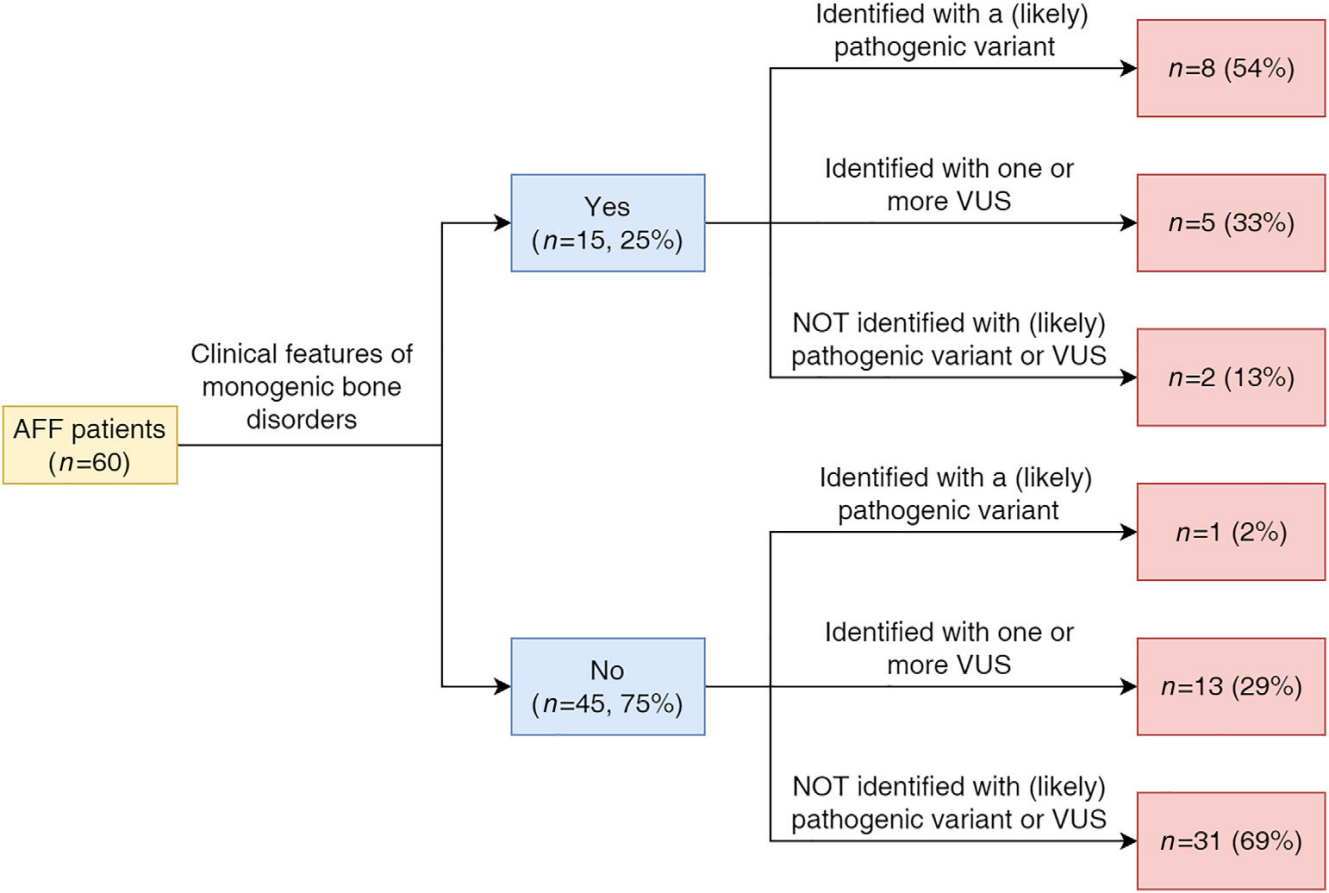
Number of atypical femur fracture (AFF) patients with clinical features of a monogenic bone disorder and with a (likely) pathogenic variant or one or more variants of uncertain significance (VUS) found by whole‐exome sequencing. The percentages in the red boxes are shown for each group: 54% of the patients with clinical features of a monogenic bone disorder were identified with a (likely) pathogenic variant.

Of the 60 AFF patients included in this cohort, 15 (25%) had clinical features of a monogenic bone disorder (Fig. 1). The phenotypes of these 15 patients are described in detail in Supplemental Table S1. Demographic and clinical characteristics of all 60 AFF patients are summarized in Table 2. The mean age of AFF onset was 62.8 years (SD = 12.7). Most patients were female (73.3%). Three patients (5%) had never used bisphosphonates, two of whom had a clinical suspicion of a monogenic bone disorder. The mean duration of bisphosphonate use was 8.6 years (SD = 4.9). Most AFF patients (*n* = 51, 85%) had been diagnosed with osteoporosis or osteopenia by dual‐energy X‐ray absorptiometry (DXA). Nine AFF patients (15%) had normal bone mineral density (BMD) by DXA, and they had all used bisphosphonates to prevent glucocorticoid‐induced osteoporosis. The proportion of patients with long‐term glucocorticoid use (>3 months oral or >1 year inhaled) was lower in the patients with a clinical suspicion of a monogenic bone disorder than in the patients without a clinical suspicion (26.7% versus 68.9%, *p* = 0.005). The other variables presented in the table were not significantly different between the patients with and without a clinical suspicion of a monogenic bone disorder.

**Table 2:** Characteristics of AFF Patients

	AFF patients with clinical suspicion of MGBD	AFF patients without suspicion for MGBD	All patients
*n* (%)	15 (25%)	45 (75%)	60 (100%)
Center (%)			
Erasmus MC	13 (86.7%)	40 (88.9%)	53 (88.3%)
LUMC	2 (13.3%)	5 (11.1%)	7 (11.7%)
Age at time of AFF (years, mean ± SD)	60.3 ± 15.9	63.6 ± 11.6	62.8 ± 12.7
Female (%)	10 (66.7%)	34 (75.6%)	44 (73.3%)
Ethnicity (%)			
African	(0.0%)	1 (2.2%)	1 (1.7%)
Arabian	(0.0%)	1 (2.2%)	1 (1.7%)
European	15 (100.0%)	40 (88.9%)	55 (91.7%)
Southern Asian	(0.0%)	3 (6.7%)	3 (5.0%)
Height (m, mean ± SD)	1.66 ± 0.11	1.63 ± 0.10	1.63 ± 0.10
Weight (kg, mean ± SD)	78.0 ± 20.1	74.2 ± 18.8	75.1 ± 19.0
BMI (kg/m^2^, mean ± SD)	27.9 ± 5.2	27.9 ± 5.7	27.9 ± 5.5
BP use before AFF (%)			
Yes	13 (86.7%)	44 (97.8%)	57 (95.0%)
No	2 (13.3%)	1 (2.2%)	3 (5.0%)
Duration of BP use among BP users (years, mean ± SD)	7.4 ± 5.4	9.0 ± 4.7	8.6 ± 4.9
Long‐term GC use (%)^a^ ^,^*			
Yes	4 (26.7%)	31 (68.9%)	35 (58.3%)
No	11 (73.3%)	14 (31.1%)	25 (41.7%)
Osteoporosis/osteopenia (%)			
Yes	14 (93.3%)	37 (82.2%)	51 (85%)
No	1 (6.7%)	8 (17.8%)	9 (15%)
Bilateral AFFs (%)			
Yes	9 (60.0%)	22 (48.9%)	31 (51.7%)
No	6 (40.0%)	23 (51.1%)	29 (48.3%)

Abbreviation: BMI = body mass index; BP = bisphosphonates; Erasmus MC = patients were included at the Bone Center of Erasmus Medical Center; GC = glucocorticoids; LUMC = patients were included at the Center for Bone Quality at Leiden University Medical Center; MGBD = monogenic bone disorder.

*Note*: Variables were compared between patients with clinical suspicion of MGBD and patients without clinical suspicion of MGBD. Categorical variables were compared by chi‐square test; in case of any cell count <5 in a contingency table, Fisher's exact test was performed. Continuous variables were compared by a simple *F* test. Missing values were excluded from the tests. *p* < 0.05 is considered significant. Variables significantly different between the two groups are marked with *.

^a^ Long‐term GC use is defined as >1 year for inhaled GC and >3 months for oral GC.

### Genetic findings regarding monogenic bone disorders in AFF patients

We examined variants identified by WES in the 37 candidate genes associated with monogenic bone disorders (Supplemental Table S2). After variant filtering, we identified 57 variants in 36 patients (60%). Among patients with a clinical suspicion of a monogenic bone disorder, 8 (54%) had a (likely) pathogenic variant and 5 (33%) had one or more VUS (Supplemental Table S3; Fig. 1). Among patients without a clinical suspicion of a monogenic bone disorder, 1 (2%) had a (likely) pathogenic variant and 13 (29%) had one or more VUS (Supplemental Table S4; Fig. 1).

### AFF patients with (likely) pathogenic variants

In total, 9 AFF patients (15%) had a likely pathogenic or pathogenic variant in the candidate genes, including 8 patients with a clinical suspicion of a monogenic bone disorder and 1 without.

### Findings in patients with a clinical suspicion of a monogenic bone disorder

As shown in Table 3, five of these likely pathogenic variants were already identified by genetic testing conducted before this study and were confirmed by the WES performed in this study. These encompass two different single‐base‐pair deletions in *PLS3* in 2 patients (S1 and S2) diagnosed with X‐linked osteoporosis, loss‐of‐function variants in *ALPL* in 2 patients (S3 and S5) diagnosed with HPP, and a nonsense variant located in the C‐propeptide‐encoding region of *COL1A2* in a patient (S4) diagnosed with Ehlers‐Danlos syndrome (EDS) arthrochalasia type (Table 3). Four of these diagnoses were only made after the occurrence of their AFF. The details of the genetic testing conducted before this study are presented in Supplemental Table S1.

**Table 3:** Identified (Likely) Pathogenic Variants in 9 AFF Patients (8 With and 1 Without Clinical Suspicion of Monogenic Bone Disorder)

ID	Sex	BP use	Diagnosis/suspicion	Diagnosis after AFF	Variant	CADD score	ACMG classification
S1	M	Yes	X‐linked osteoporosis	Yes	*PLS3*, c.842delT, p.Leu281fs, heterozygous	N/A	Pathogenic
S2	M	Yes	X‐linked osteoporosis	Yes	*PLS3*, c.235delT, p.Tyr79fs, heterozygous	N/A	Pathogenic
S3	F	Yes	Hypophosphatasia	Yes	*ALPL*, c.997 + 2 T > A, p.?, heterozygous	16	Pathogenic
S4	F	Yes	Ehlers‐Danlos syndrome (EDS) arthrochalasia type 2	Yes	*COL1A2*, c.3973G > T, p.Gly1325*, heterozygous	44	Likely pathogenic
S5	F	No	Hypophosphatasia	No	*ALPL*, c.201_203delGAC, p.Thr68del, heterozygous	N/A	Likely pathogenic
S6^a^	F	Yes	Monogenic osteoporosis	N/A	*COL1A2*, c.964G > A, p.Gly322Ser, Heterozygous	26	Likely pathogenic
S7^a^	F	Yes	Monogenic osteoporosis	N/A	*COL1A2*, c.964G > A, p.Gly322Ser, heterozygous	26	Likely pathogenic
S15	F	Yes	Monogenic osteoporosis	N/A	*LRP5*, c.2827 + 1G > A, p.?, heterozygous	22	Likely pathogenic
NS2	F	Yes	None	N/A	*TCIRG1*, c.2008C > T, p.Arg670*, heterozygous	39	Likely pathogenic

Abbreviation: ACMG = American College of Medical Genetics and Genomics; AFF = atypical femur fracture; BP = bisphosphonates; CADD = Combined Annotation Dependent Deletion.

*Note*: The complete list of rare variants identified for these patients including variants classified as uncertain significance and (likely) benign and gene transcripts are indicated in Supplemental Tables S3 and S4.

^a^ Indicates siblings.

Three patients (S6, S7 [siblings], and S15) were not genetically tested during clinical practice. A missense variant (glycine substitution in the triple‐helical domain) in *COL1A2* classified as likely pathogenic was identified in the two siblings (S6 and S7). These two patients had severe osteoporosis and multiple fractures but no other signs of OI or EDS. A heterozygous splicing variant in *LRP5* (c.2827 + 1G > A) classified as likely pathogenic was identified in patient S15. This patient had an incomplete AFF at the right side when she was 80 years old after 9 years of alendronate treatment. She had multiple vertebral fractures at the age of 53 years and low BMD. Later, she also had multiple nonvertebral fractures, and she lost 7 cm in height due to vertebral fractures. Both her mother and aunt also had lost height at an older age.

### Findings in patients without a clinical suspicion of monogenic bone disorders

Among the 46 patients without a clinical suspicion of monogenic bone disorders, one (NS2) was identified with a heterozygous c.2008C > T (p.Arg670*) variant in *TCIRG1*, a gene responsible for autosomal recessive osteopetrosis (Table 3). This variant was classified as likely pathogenic as it leads to a new stop codon that could result in shortened protein or nonsense‐mediated mRNA decay (NMD). It has also been reported to be pathogenic in all seven submissions to ClinVar, but no experimental evidence was provided. This patient had low‐normal BMD at the hip (*T*‐score = −1.2 SD both before and around the time of AFF). *T*‐score at the lumbar spine was increased (+3.3 SD) possibly in part attributable to degenerative changes. She received bisphosphonates because of the use of aromatase inhibitors and was not known to have bone metastases.

Among the AFF patients identified with likely pathogenic variants, one patient diagnosed with HPP was never exposed to bisphosphonates (S5). The other patients were treated with bisphosphonates, including one patient who was later diagnosed with HPP (S3).

### AFF patients with variants classified as uncertain significance

In total, 18 (30%) AFF patients had one or more variants classified as VUS. We listed 12 AFF patients carrying a VUS with a CADD score **≥**20 or had an indel, for which no CADD score was available, in Table 4. All of these patients had been exposed to bisphosphonates. Four of them (S8–S11) were suspected of a monogenic bone disorder. Patients S8 and S11, who were suspected of OI, had a 3‐base‐pair deletion in *COL1A1* and a missense variant c.2933G > A (p.Arg978His) in *COL1A2*, respectively. Patient S9 was suspected of monogenic osteoporosis and had a missense variant c.3220G > T (p.Val1074Phe) in *LRP5*. Patient S10 had a heterozygous VUS in *BMP1*, a gene responsible for autosomal recessive OI. The clinical phenotypes of these patients are listed in Supplemental Table S1.

**Table 4:** Identified Variants Classified as Uncertain Significance and With a CADD Score ≥ 20 in AFF Patients

ID	Sex	BP use	Diagnosis/suspicion	Variant	CADD score	ACMG classification
S8	F	Yes	Osteogenesis imperfecta	*COL1A1*, c.206_208delTGT, p.Leu69del, heterozygous	N/A	Uncertain significance
S9	F	Yes	Monogenic osteoporosis	*LRP5*, c.3220G > T, p.Val1074Phe, heterozygous	20	Uncertain significance
S10	M	Yes	Monogenic osteoporosis	*BMP1*, c.2134G > A, p.Gly712Ser, heterozygous	33	Uncertain significance
S11	F	Yes	Osteogenesis imperfecta	*COL1A2*, c.2933G > A, p.Arg978His, heterozygous	25	Uncertain significance
NS2	F	Yes	None	*P4HB*, c.874G > A, p.Asp292Asn, heterozygous	30	Uncertain significance
				*TNFRSF11A*, c.1618A > G, p.Met540Val, heterozygous	22	Uncertain significance
NS3	F	Yes	None	*CREB3L1*, c.803G > A, p.Arg268Gln, heterozygous	35	Uncertain significance
NS4	F	Yes	None	*TCIRG1*, c.2216C > T, p.Ala739Val, heterozygous	32	Uncertain significance
NS5	F	Yes	None	*SERPINH1*, c.266C > T, p.Thr89Met, heterozygous	29	Uncertain significance
NS6	F	Yes	None	*PLOD2*, c.587C > T, p.Thr196Ile, heterozygous	26	Uncertain significance
NS7	M	Yes	None	*LEPRE1*, c.1975C > A, p.His659Asn, heterozygous	26	Uncertain significance
NS8	F	Yes	None	*P4HB*, c.484A > G, p.Lys162Glu, heterozygous	22	Uncertain significance
NS9	F	Yes	None	*PHEX*, c.1094A > G, p.Tyr365Cys, heterozygous	20	Uncertain significance

Abbreviation: ACMG = American College of Medical Genetics and Genomics; AFF = atypical femur fracture; BP = bisphosphonates; CADD = Combined Annotation Dependent Deletion.

*Note*: Variants identified by whole‐exome sequencing are shown if classified as uncertain significance and with a CADD score ≥ 20 (or not applicable [N/A]). The complete list of rare variants identified for these patients including variants with CADD score < 20 and gene transcripts are indicated in Supplemental Tables S3 and S4.

Eight AFF patients (NS2‐9) without a clinical suspicion of a monogenic bone disorder carried one or more heterozygous VUS with a CADD score ≥ 20 in *P4HB*, *PHEX*, *TNFRSF11A*, *CREB3L1*, *TCIRG1*, *SERPINH1*, *LEPRE1*, and *PLOD2* (Table 4). Variants in *P4HB* and *PHEX* are associated with dominant bone‐related disorders, ie, Cole‐Carpenter syndrome 1 and X‐linked hypophosphatemia (XLH), respectively. Patients NS2 and NS8 both harbored a heterozygous variant in *P4HB*, namely c.874G > A (p.Asp292Asn) and c.484A > G (p.Lys162Glu), respectively. Patient NS8 had dentures at the age of 25 years and suffered one fracture of her clavicle at the age of 10 years. She also had chronic obstructive pulmonary disease (COPD) for which she only once used a short course of glucocorticoids and had had vertebral fractures at the age of 62 years. Except for fragility fractures, both NS2 and NS8 had no other clinical features of Cole‐Carpenter syndrome (ie, craniosynostosis, ocular proptosis, hydrocephalus, or distinctive facial features). Patient NS9 (female) had a heterozygous variant c.1094A > G (p.Tyr365Cys) in *PHEX* but had normal phosphate level (1.17 nmol/L, normal value 0.8–1.4) and no signs or symptoms suggestive of XLH.

### Comparison with population controls

To assess whether the AFF cohort was enriched for carriers of (likely) pathogenic variants or VUS, we analyzed 125,748 exomes from gnomAD and classified the variants using filtering criteria outlined in Supplemental Table S5. It was estimated that only 1% of controls were carriers of a (likely) pathogenic variant. When estimated using the same method, 11.8% AFF cases (excluding one of the siblings) were carriers of a (likely) pathogenic variant, which was slightly lower than the percentage resulting from classification using the ACMG guideline but still significantly higher than the percentage in the gnomAD controls (*p* < 0.0001) (Supplemental Table S5). The prevalence of individuals carrying VUS and VUS with CADD ≥20 were not different among AFF cases and gnomAD controls (*p* > 0.05) (Supplemental Tables S5 and S6).

### Copy number variation analyses

Using DNA array genotyping data, we identified a 12.7 Mb deletion (Chr 6: 71,561,194 – 84,303,230; build 37) in one patient (NS24) without a clinical suspicion of a monogenic bone disorder (Supplemental Fig. S1). In this patient, no SNV or indel was found by WES. The CNV was classified as likely pathogenic (total score = 0.9, fulfilled criteria 1A, 2A, 3C, 4C, and 5G in the ClinGen CNV Pathogenicity Calculator for Copy Number Loss). This deletion encompasses 87 genes starting with *SMAP1* up to *SNAP91*, including among others *COL12A1*, *MYO*, *LCA5*, *MRAP2*, and *HTR1B*, as well as *TENT5A* (*FAM46A*), a gene in the candidate gene list and related to autosomal recessive OI. The patient had an incomplete AFF. She was diagnosed with osteoporosis at the age of 28 years after a renal transplant at age 21 years and started bisphosphonates for 9 years until the AFF occurred at age 36 years. She was reported to have congenital abnormalities of the musculoskeletal system, as well as intellectual disability, obesity, hypertension, renal insufficiency, hypermetropy, and deafness. In addition to an AFF, she had suffered from multiple fractures, including rib fractures and ankle and knee fractures.

## Discussion

Previous studies have suggested that AFFs are associated with monogenic bone disorders.^(^ ^13^, ^15^ ^)^ This is further supported by the current findings where, out of 60 AFF patients, 25% had clinical features consistent with a monogenic bone disorder and 15% were found to harbor a (likely) pathogenic variant in one of the 37 known candidate genes, including one patient who was a carrier of a variant for an autosomal recessive bone disorder. In the research setting, the yield of (likely) pathogenic variants in those clinically suspected of a monogenic bone disorder (54%) was much higher than in patients who were not (2%). This emphasizes the importance of a thorough clinical evaluation and genetic testing in AFF patients suspicious for a potential underlying monogenic bone disorder.

We estimated 1% of the population controls from gnomAD carried a (likely) pathogenic variant. Previous studies have reported 1% to 6.2% of the general population to have ACMG clinically actionable variants, in which other and more genes were studied,^(^ ^42^, ^43^, ^44^, ^45^, ^46^ ^)^ suggesting a background level of (likely) pathogenic variants in seemingly healthy individuals. However, the enrichment of pathogenic variants in monogenic bone disorder genes in AFF patients cannot be explained merely by chance alone.

### Variants classified as likely pathogenic in AFF patients

Eight AFF patients with a clinical suspicion of a monogenic bone disorder had a (likely) pathogenic variant in one of the 37 candidate genes. Among them, one of the two patients with X‐linked osteoporosis and a variant in *PLS3* was published as a case report^(^ ^47^ ^)^ and summarized in a review.^(^ ^13^ ^)^ Also, a patient was diagnosed with EDS arthrochalasia type with an aneurysm in the aorta, multiple fractures after low‐energy trauma, and extensibility of the joints, but the patient also had other symptoms that overlap with OI, including blue sclerae, low BMD, hyperkyphosis, and dental prosthesis at young age.^(^ ^15^ ^)^ Therefore, this patient could also be classified as having OI/EDS overlap or “COL1‐related overlap disorder” as described by Silvia Morlino and colleagues.^(^ ^48^ ^)^ The likely pathogenic variant in this patient is located near the C‐terminal end of *COL1A2*, which may escape nonsense‐mediated mRNA decay (NMD) and produce an altered C‐propeptide domain,^(^ ^49^ ^)^ so its actual function remains to be elucidated by functional experiments.

Two additional (likely) pathogenic variants were identified in three AFF patients with a suspicion of monogenic osteoporosis who had not been genetically tested in a clinical setting. Two siblings had a variant in COL1A2, previously reported in OI patients (dominant form).^(^ ^50^, ^51^, ^52^ ^)^ Variants in *COL1A2* have been repeatedly linked to AFF.^(^ ^15^ ^)^ The other patient had a likely pathogenic variant in *LRP5*, previously reported in an OPPG patient whose mother was a carrier with moderately reduced bone mass.^(^ ^53^ ^)^ In fact, multiple studies have shown that carriers of a heterozygous variant in this gene also had reduced bone mass.^(^ ^54^, ^55^ ^)^

In the patient who had a likely pathogenic variant in *TCIRG1*, the pertinence of this variant to AFF is unclear. A de novo missense variant in *TCIRG1* has been reported in a 3‐year‐boy diagnosed with autosomal dominant osteopetrosis.^(^ ^56^ ^)^ However, since parents of other osteopetrosis patients carrying the same variant were healthy, a dominant effect of a single pathogenic variant in *TCIRG1* on bone quality is yet unclear.^(^ ^57^ ^)^

### Variants classified as uncertain significance in AFF patients

We cannot make conclusions on the relevance of the identified VUS because many of them were in genes responsible for recessive diseases and the prevalence of VUS carriers estimated from WES data of gnomAD controls did not suggest enrichment of VUS in AFF cases. Notably, three patients had VUS in *COL1A1*, *LRP5*, and *COL1A2* that matched their suspected monogenic bone disorders. The VUS in *COL1A2* has been reported in two Malaysian OI patients, one of whom had two different variants in this gene.^(^ ^58^ ^)^ The pathogenicity of these variants cannot be determined without further evidence, such as a functional study of the variant and family phenotypes.

### Copy number variation in an AFF patient

We identified a likely pathogenic CNV, which encompasses *TENT5A* (*FAM46A*), but its pertinence to the phenotype of the patient and to AFF remains uncertain. The patient experienced non‐AFF fractures that could also be secondary to her other conditions, such as chronic kidney disease, and immunosuppressants for renal transplantation. Deletions in this region have been associated with the 6q12‐14.3 deletion syndrome, involving a diversity of clinical features that matched the phenotype of our patient such as intellectual disability, congenital deformities, cardiovascular and renal abnormalities, hearing loss, and hypotonia, but not limb deformities or fragility fractures.^(^ ^59^, ^60^, ^61^ ^)^ *TENT5A* is responsible for autosomal recessive OI, but haploinsufficiency has not been documented as one of its dysfunction mechanisms and our patient lacked other signs of OI.^(^ ^62^ ^)^ Van Esch and colleagues reported three patients with 6q deletion syndrome and developmental delay, mild dysmorphism, and signs of lax connective tissue, sharing a 3.7 Mb deleted region overlapping with that in our patient, which includes *COL12A1*.^(^ ^63^ ^)^ Despite a complete knockdown of this gene causing bone fragility in mice,^(^ ^64^ ^)^ pathogenic variants in *COL12A1* are associated with recessive Ullrich congenital muscular dystrophy 2 (MIM 616470), dominant Bethlem myopathy 2 (MIM 616471), or myopathic EDS,^(^ ^65^ ^)^ but not fractures.

### Proposed pathogenesis of AFFs related to monogenic bone disorders

Several factors have been proposed contributing to AFF occurrence, such as degree of femoral bowing,^(^ ^66^, ^67^ ^)^ femur size,^(^ ^68^ ^)^ use of glucocorticoids,^(^ ^69^ ^)^ use of bisphosphonates^(^ ^70^ ^)^ and other antiresorptive drugs like denosumab,^(^ ^71^ ^)^ and genetic factors.^(^ ^12^, ^13^ ^)^ Particularly, prolonged bisphosphonate use has been associated with an increased risk of AFFs.^(^ ^5^ ^)^

However, AFFs also occur in bisphosphonate‐naïve patients, precluding bisphosphonates being a prerequisite for AFF.^(^ ^13^, ^15^ ^)^ In our current AFF cohort, three patients (including two suspected of a monogenic bone disorder) had never been exposed to bisphosphonates. Except one diagnosed with HPP with an ALPL variant, the other two patients did not have a (likely) pathogenic variant in the 37 candidate genes, but the existence of a novel genetic cause for a monogenic bone disorder cannot be excluded.

Rare genetic variants associated with monogenic bone disorders may increase AFF risk for several reasons, including compromised femoral bone quality, impaired microcrack repair, or deformities of the femur.^(^ ^70^, ^72^ ^)^ These disorders involve primary defects in bone mineralization, remodeling, collagen synthesis/structure, and osteocyte function.^(^ ^13^ ^)^ They may interact with the effect of antiresorptive drugs depending on the type of bone disorder. Although these drugs may affect patients with primary osteoclast defects more, those with primary osteoblast defects may also be affected because normally bone resorption and formation are coupled. Further studies are needed to elucidate how these rare genetic variants contribute to AFF and/or interact with bisphosphonates.

### Implications for clinical practice

Our study has several implications for clinical practice. Fifteen percent of AFF patients in our cohort had (likely) pathogenic variants identified by WES, indicating unrecognized monogenic bone disorders. Clinicians should examine AFF patients for features of monogenic bone disorders, such as signs of OI, and decreased serum alkaline phosphatase or serum phosphate concentrations for HPP and chronic hypophosphatemia, respectively. Depending on patient wishes, genetic screening may be performed after clinical suspicion. Mineralization disorders should be excluded through clinical and lab examination before starting bisphosphonates when confirming a diagnosis through genetic testing has relevance not only for counseling but also for medical management. Notably, our cohort is enriched with patients with previously unrecognized OI and other forms of monogenic bone disorders such as X‐linked osteoporosis. Most presented with AFFs after being treated with bisphosphonates. Despite bisphosphonates being used to prevent fragility fractures in these patients, there is little evidence for their effectiveness in these rare diseases. It is important to further investigate whether the risk of AFF is high because of the underlying monogenic disease or because of (long‐term) use of bisphosphonates or a combination.

### Limitations

This study is the first comprehensive evaluation of rare genetic variants relating to monogenic bone disorders in a relatively large AFF cohort. We acknowledge several limitations. Recruiting from specialist centers for complex and rare calcium and bone disorders may have resulted in an overrepresentation of patients with an underlying monogenic bone disorder. Moreover, subjectivity cannot be avoided in evaluation of a potential monogenic bone disorder, especially when secondary factors are present together with clinical signs of genetic cause and also clinical experience plays a role. Variants classified as likely pathogenic, with no prior report or functional data, require confirmation of pathogenesis, as well as heterozygous variant in a gene where haploinsufficiency is not a pathogenic mechanism, such as *TCIRG1*. WES may miss certain genetic variants, such as variants in regulatory or intronic regions, other forms of structural variants than CNVs, and variants in unidentified monogenic bone disorder genes. Polygenic risk factors (involving common genetic variants) might also contribute to the risk of AFFs, but this is beyond the scope of this study.

In summary, the findings from this study strongly suggest that genetic variants associated with monogenic bone disorders play a role in the pathogenesis of AFF, although the underlying mechanism is still unclear. A high yield (54%) of identified likely pathogenic variants by WES in the AFF patients with a clinical suspicion of monogenic bone disorders highlights the relevance of thorough clinical evaluation and genetic testing in AFF patients for clinical management, family counseling, and reproductive decisions. Because this is the first study to determine the prevalence of monogenic bone disorders in an AFF cohort from specialist bone centers, the current findings should be investigated in other cohorts.

## Author Contributions

**Wei Zhou:** Data curation; formal analysis; investigation; methodology; software; visualization; writing – original draft; writing – review and editing. **Jeroen GJ van Rooij:** Methodology; supervision; writing – review and editing. **Denise M van de Laarschot:** Data curation; funding acquisition; project administration; writing – review and editing. **Zografia Zervou:** Project administration; writing – review and editing. **Hennie Bruggenwirth:** Writing – review and editing. **Natasha M Appelman‐Dijkstra:** Project administration; resources; writing – review and editing. **Peter R Ebeling:** Writing – review and editing. **Serwet Demirdas:** Writing – review and editing. **Annemieke JMH Verkerk:** Methodology; supervision; writing – review and editing. **M Carola Zillikens:** Conceptualization; funding acquisition; investigation; methodology; project administration; resources; supervision; writing – review and editing.

## Conflicts of Interest

PRE has received a research grant from Amgen and his institute received honoraria from Amgen. The remaining authors have no conflicts of interest to declare.

### Peer Review

The peer review history for this article is available at https://www.webofscience.com/api/gateway/wos/peer‐review/10.1002/jbmr.4801.

## Supporting information


**Supplemental Table S1.** The Phenotypes of the Patients Suggesting a Monogenic Bone Disorder
**Supplemental Table S2.** Candidate Genes Associated With Monogenic Bone Disorders
**Supplemental Table S3.** Genetic Findings in AFF Patients With a Clinical Suspicion of Monogenic Bone Disorders
**Supplemental Table S4.** Genetic Findings in AFF Patients Without a Clinical Suspicion of Monogenic Bone Disorders
**Supplemental Table S5.** Comparison Between Genetic Findings in 59 AFF Cases and 125,748 gnomAD Controls
**Supplemental Table S6.** VUS Identified From Supplemental Table S5 Grouped by CADD Score

**Supplemental Fig. S1.** (*A*) Detected copy number variation (heterozygous deletion) on chromosome 6q found in patient NS24 displayed in Nexus software (Biodiscovery). (*B*) Genomic region of the detected CNV adapted from UCSC genome browser (hg19), showing genes in the involved region. The deletion region in the patient is indicated in red.

## Data Availability

Sharing raw or processed individualized sequencing results of the patients are not allowed due to General Data Protection Regulation (GDPR). Requests to access the datasets should be directed to MCZ, m.c.zillikens@erasmusmc.nl.
