# Supplementary figures and images for: Prevalence of Monogenic Bone Disorders in a Dutch Cohort of Atypical Femur Fracture Patients
Source: J Bone Miner Res. 2023 Apr 19;38(6):896–906. doi: 10.1002/jbmr.4801 (PMC10946469; doi:10.1002/jbmr.4801)

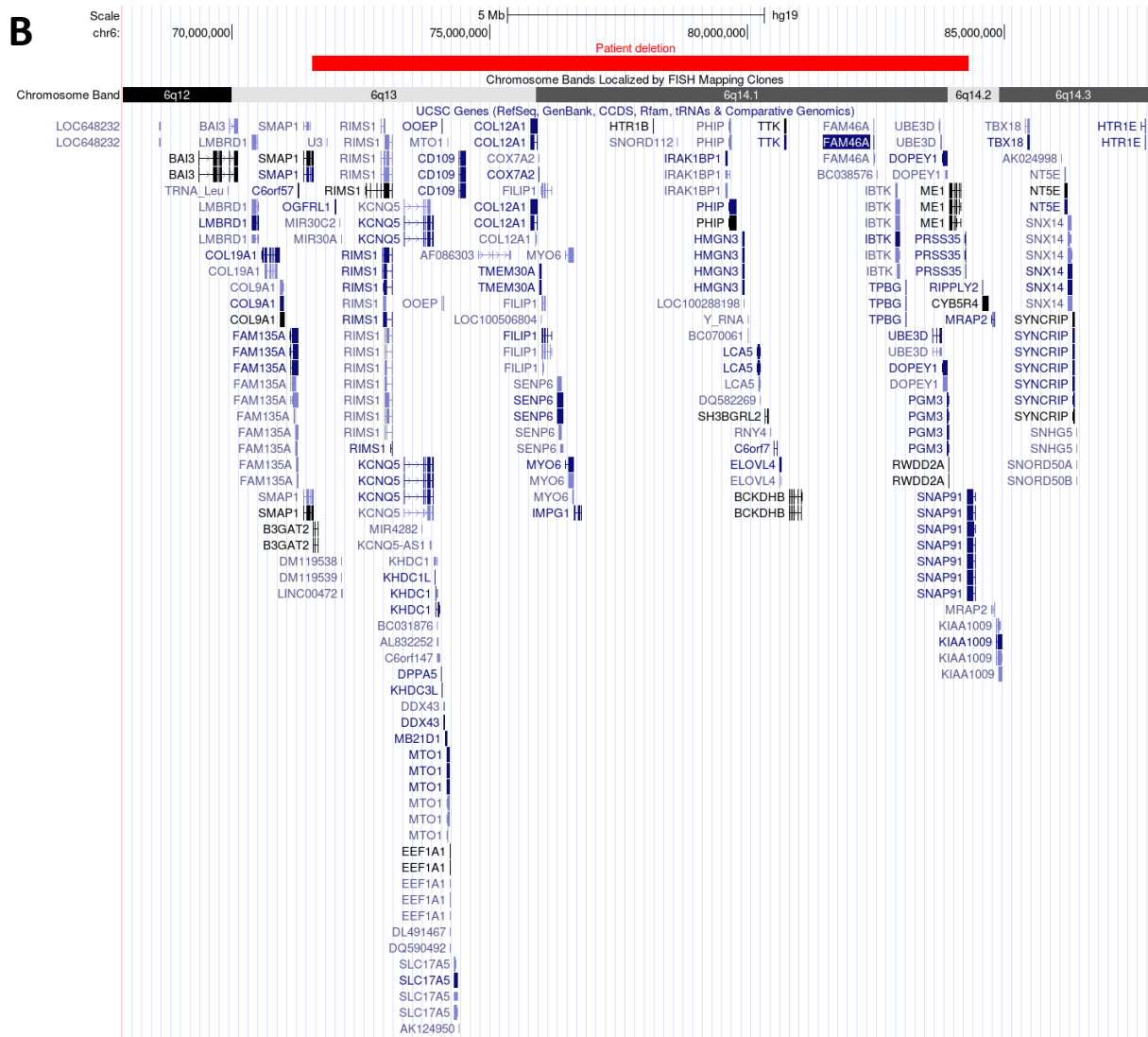

Supplement: Supplementary file 2 — Supplemental Fig. S1. (A) Detected copy number variation (heterozygous deletion) on chromosome 6q found in patient NS24 displayed in Nexus software (Biodiscovery). (B) Genomic region of the detected CNV adapted from UCSC genome browser (hg19), showing genes in the involved region. The deletion region in the patient is indicated in red. [file JBMR-38-896-s001.pdf]
